# A Non-Hereditary Case of Hereditary Leiomyomatosis and Renal Cell Carcinoma Syndrome

**DOI:** 10.7759/cureus.13344

**Published:** 2021-02-15

**Authors:** Vaishali Kapila, Arjun G Kalra, David L Stockman

**Affiliations:** 1 Internal Medicine, Central Michigan University College of Medicine, Mt. Pleasant, USA; 2 Internal Medicine, Brooke Army Medical Center, San Antonio, USA; 3 Pathology, OrthoPath Labs, Kalamazoo, USA

**Keywords:** chromophobe renal carcinoma, papillary rcc, reed syndrome, leiomyoma, autosomal dominant, fumarate hydratase, krebs cycle

## Abstract

Hereditary leiomyomatosis and renal cell carcinoma (HLRCC) syndrome is believed to result from an autosomal dominant mutation in the fumarate hydratase (FH) gene on chromosome 1. It is characterized by leiomyomas, mainly uterine or cutaneous, and renal cell carcinoma (RCC). The most common type of RCC associated with HLRCC is type II papillary RCC although other types are seen. Of note, chromophobe RCC has not been described in previously documented cases of HLRCC. HLRCC is typically associated with germline mutations with occasional somatic mutations reported, however, to the best of our knowledge, none have yielded the full phenotype until now. Herein, we report a case of a 45-year-old woman who underwent a hysterectomy following a year of heavy vaginal bleeding, yielding a diagnosis of uterine leiomyomas. Eight months later, the patient presented with hematuria and was subsequently found to have a left renal mass. Following a left radical nephrectomy, histologic exam revealed a chromophobe RCC with FH deficiency.

## Introduction

Hereditary leiomyomatosis and renal cell carcinoma (HLRCC) syndrome or Reed syndrome, results from an autosomal dominant mutation in fumarate hydratase (FH) [1]. Patients possessing these mutations are more susceptible to developing the phenotypic presentation of multiple uterine and/or cutaneous leiomyomas in addition to renal cell carcinoma (RCC) [2]. Demographically, the great majority of patients diagnosed with HLRCC are female due to the commonality of uterine leiomyomas. However, cases of HLRCC have also been seen in males with cutaneous leiomyomas occurring on their chest, back, head, and/or upper extremities [3].

Histologically, all leiomyomas seen in HLRCC exhibit typical smooth muscle histology as well as some unique features including fascicles of fusiform spindle cells with eosinophilic cytoplasm, nuclear atypia, and mitotic activity. Importantly, the leiomyomas lack necrosis [4]. Diagnosed patients with HLRCC are predisposed to the development of RCCs. Most commonly, HLRCC patients develop type II papillary carcinomas but other types have been reported: undifferentiated papillary carcinoma, conventional RCC, a renal cyst, and now chromophobe, as presented in this case [5-7].

Genetically, these patients are characterized by mutations in fumarate dehydratase, which serves as a tumor suppressor and produces an enzyme important to the citric acid cycle [4]. Functionally, the enzyme converts fumarate to L-malate during Krebs cycle progression, and alteration of its functionality changes the metabolic capacity of the cell [8]. Numerous mutations in the FH gene have been reported in numerous databases [1]. In HLRCC patients, mutations have been predominantly germline with some showing somatic mutations [9]. Most mutations are heterozygous as homozygous mutations of FH cause complete deficiency and severe metabolic problems resulting in death before ten years of age [10]. 

Cancer in HLRCC is often aggressive and when found in a higher stage, mortality rates increase, resulting in poor outcomes. Patients and family members would benefit from early identification and screening, however, due to its rarity, HLRCC is not often thought about and increased awareness is necessary. Herein, we present a case of a 45-year-old female with a history of uterine leiomyomas who was found to have chromophobe RCC that was FH deficient with no family history, a case not previously described in the literature.

## Case presentation

**Figure 1:**
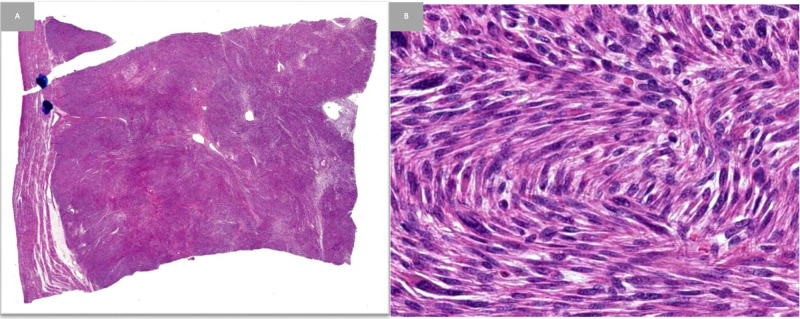
Low and high power views of uterine leiomyoma A, A low power image demonstrates a well-circumscribed lesion; B, Higher power shows fascicles of spindled smooth muscle cells in the leiomyoma. Note the lack of atypia, necrosis, and mitotic activity

A 45-year-old Asian-Indian female presented to her physician with the symptoms of constant fatigue, pain, inability to focus, and heavy vaginal bleeding every two weeks that would last 8-10 days and remit. The patient reported that this pattern occurred for a year before it became constant light bleeding that did not cease. She was found to have an iron deficiency anemia, not corrected by oral iron supplementation. She had no history of smoking and only drank a couple of times a month. There was no known family history of leiomyomas or RCC. Upon completion of pelvic ultrasound, two uterine leiomyomas were detected with possible endometrial cavity involvement. The largest leiomyoma was observed to be 3.4 cm in the greatest dimension on ultrasound. The patient then underwent a laparoscopic complete hysterectomy about a year and a half after the onset of symptoms. Gross observation of the removed uterus revealed an 11 x 9 x 7 cm uterus with two fibroids, an endometrial polyp, and unremarkable tubes and ovaries. Histologically, the leiomyomas demonstrated classic benign histologic findings (Figure 1). The patient had no symptomology or complications until eight months later when the patient presented with hematuria. 

**Figure 2:**
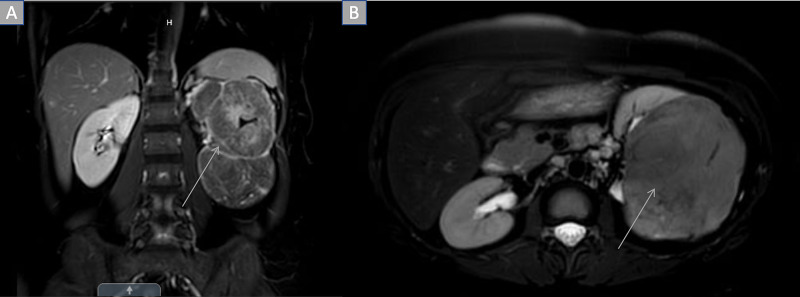
MRI abdomen demonstrating left renal mass A: Coronal view of exophytic left renal mass; B: Transverse view of left renal mass.

**Figure 3:**
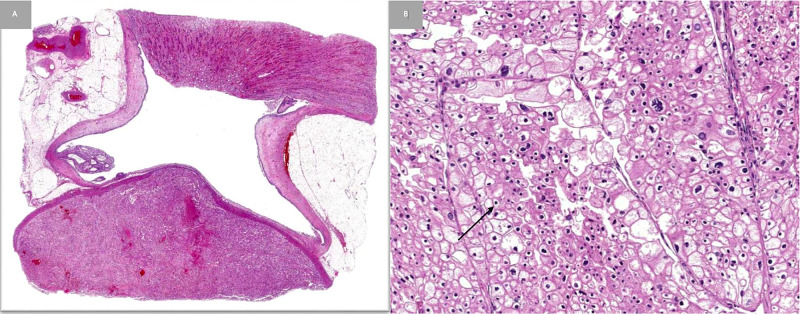
Low (A) and high (B) power views of chromophobe renal cell carcinoma (RCC) A: Low power view of the chromophobe RCC; B: High power view of chromophobe RCC with typical eosinophilic cytoplasm, distinct cell membranes, and perinuclear clearing (arrow).

**Figure 4:**
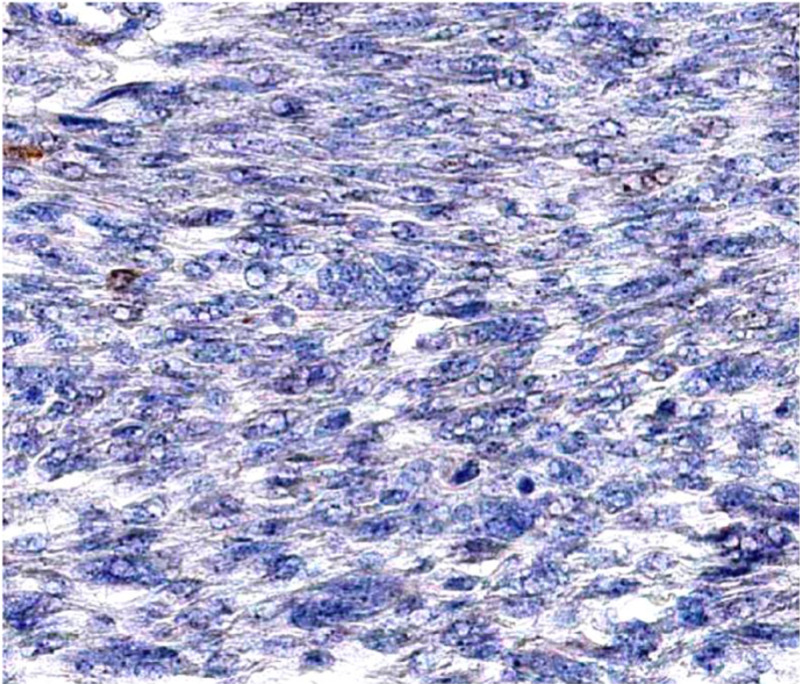
Immunochemical staining of renal cell carcinoma demonstrating loss of nuclear staining for fumarate hydratase

**Figure 5:**
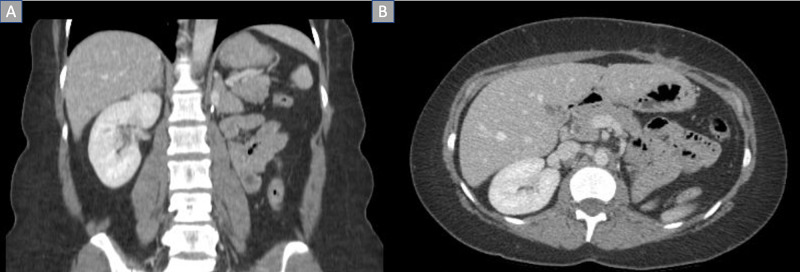
CT abdomen post left radical nephrectomy Surveillance CT abdomen demonstrating left nephrectomy with no residual disease in (A) coronal view and (B) transverse view.

X-ray, computed tomography (CT), and magnetic resonance imaging (MRI) were performed and revealed a left enhancing, exophytic renal mass measuring 14 x 11 x 9.3 cm (Figure 2). Positron emission tomography (PET) CT revealed no focus of increased metabolism concerning for metastatic disease. The patient underwent a transabdominal open left radical left nephrectomy with the removal of the left adrenal gland and hilar and periaortic lymph node dissection. The mass removed measured 12 x 12 x 8 cm. Gross examination of the kidney specimen revealed multiple sites of necrosis with extension of the tumor to the renal sinus, ureter, and segmental branches of the renal vein without invasion. The tumor did not invade the Gerota’s fascia. Histological examination revealed chromophobe RCC due to the presence of cells with eosinophilic cytoplasm and a halo around the nucleus (Figure 3). All 12 lymph nodes histologically assessed were benign. Staining for FH was negative (Figure 4). The patient was staged with a pT3aMxN0 disease. Five years post nephrectomy, the patient reports no worrisome symptoms and shows no signs of metastatic disease (Figure 5).

## Discussion

Our case deviates significantly from previously described cases in the fact that the patient had no concerning family history to raise suspicion of HLRCC and that her RCC was chromophobe, which has not previously been described in association with HLRCC.

HLRCC does not have uniform clinical management due to its rarity. It is characterized by leiomyomas, mainly in the uterus or skin, and RCC. It is believed to be due to an autosomal dominant mutation in the FH gene, however, there are cases where it is not always present [1]. As a result of the autosomal dominant inheritance, some families have a strong history of HLRCC [11]. Classically, the syndrome impacts females, with a minority of cases occurring in males [11]. Females with HLRCC are found to have uterine leiomyomas at a much younger age compared to women who do not have HLRCC [12]. Leiomyomas typically occur before the RCC and can occur in other locations, with the skin being the most common [9]. The reported average age for women to develop uterine leiomyomas is 38 [13]. However, the reported average age of HLRCC patients to present with uterine leiomyomas is 28-32 years old [12].

The most commonly diagnosed sporadic RCC is clear cell carcinoma with it representing 70%-75% of cases [14]. Papillary RCCs represent about 15% of RCCs, but in HLRCC, type II papillary carcinoma is most commonly seen [14]. Most cases present at a late stage, highlighting the need for screening in patients with HLRCC. Other types of renal masses seen in HLRCC patients include clear cell, and renal cysts [15]. While the average age of RCC in non-HLRCC patients is around 64 years old, HLRCC patients are diagnosed with RCC at much younger ages around 44 years old [16,17]. As previously mentioned, the chromophobe subtype has not been previously described in association with HLRCC, making this a unique case. Recognition of HLRCC being implicated in a wide variety of RCC subtypes can better help maintain an appropriate level of suspicion, thus possibly helping to initiate earlier screening and testing in family members to improve outcomes for this patient population.

Outcomes seem to depend on the diagnosis of the RCC of a patient rather than the leiomyomas. This is especially important given the aggressive nature of type II papillary RCC connected to HLRCC. Whether the RCC is of sporadic or HLRCC origin, the drug, sunitnib, is frequently involved in the treatment of advanced disease due to its ability to block vascular endothelial growth factor (VEGF) pathways, which are frequently implicated in the pathogenesis of RCCs [18]. While somatic mutations are sparsely mentioned throughout the literature, they had not manifested the entire phenotype, limiting its clinical relevance until now [9]. By demonstrating FH deficiency histologically in the absence of any family history, we have shown that a somatic mutation can yield non-hereditary HLRCC. To better help identify vulnerable populations, a case can be made about screening renal cell cancers histologically for FH deficiency. Additionally, a better understanding of the pathogenesis of HLRCC may yield more insight into management.

## Conclusions

It is important to study HLRCC as a result of the aggressiveness of the RCC it can cause. Being able to find more commonalities among cases will help identify at-risk families and hopefully prevent RCC or catch it in an earlier stage. While the RCC typically manifests as type II papillary, increasing amounts of literature are showing that other subtypes are implicated in the syndrome. Until now, there have been no identified cases of chromophobe RCC being implicated in HLRCC. Additionally, while the vast majority of cases are hereditary, our case demonstrating FH deficiency with no other family history suggests a somatic mutation. Until now, it was not known that a somatic mutation could yield a full phenotype. This brings to light a reasonable argument for screening RCCs for the FH mutation. Whether the mutation is germline or somatic, testing subsequent generations of family members to prevent deleterious outcomes is valuable.
